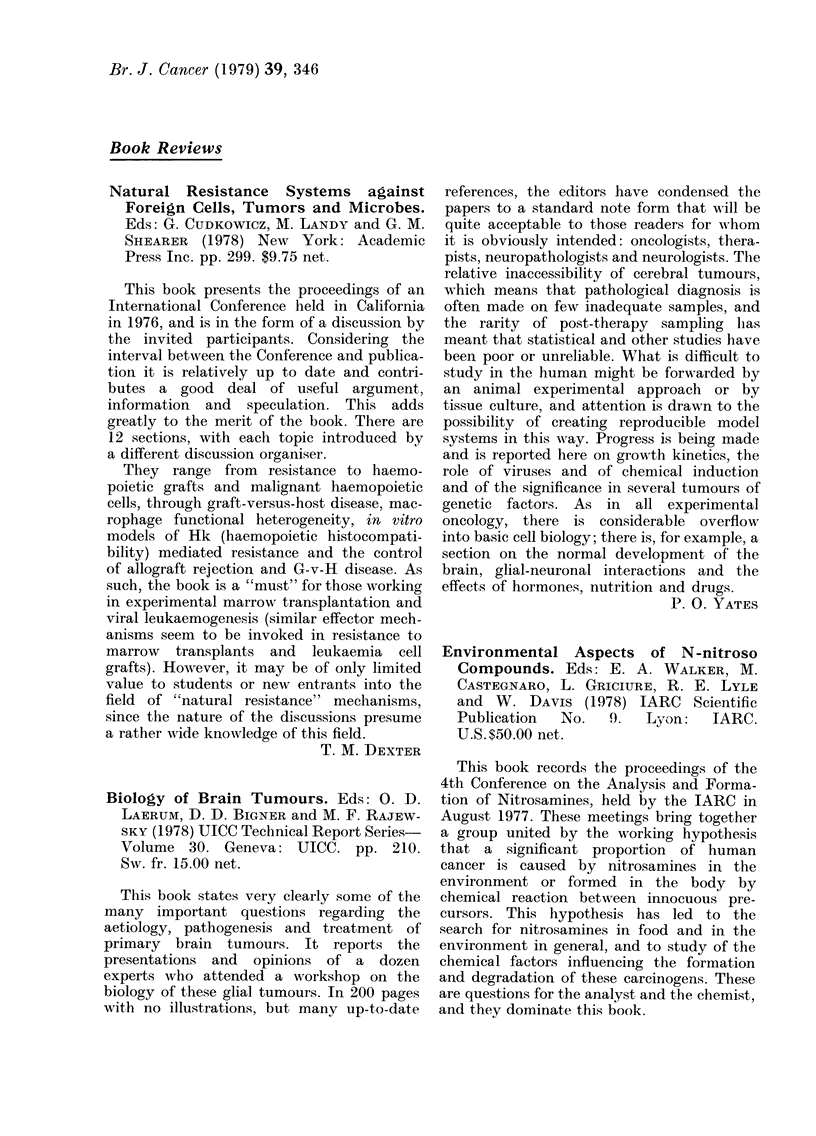# Natural Resistance Systems against Foreign Cells, Tumors and Microbes

**Published:** 1979-03

**Authors:** T. M. Dexter


### Book Reviews

**Natural Resistance Systems against Foreign Cells, Tumors and Microbes.** Eds: G. Cudkowicz, M. Landy and G. M. Shearer (1978) New York: Academic Press Inc. pp. 299. $9.75 net.

This book presents the proceedings of an International Conference held in California in 1976, and is in the form of a discussion by the invited participants. Considering the interval between the Conference and publication it is relatively up to date and contributes a good deal of useful argument, information and speculation. This adds greatly to the merit of the book. There are 12 sections, with each topic introduced by a different discussion organiser.

They range from resistance to haemopoietic grafts and malignant haemopoietic cells, through graft-versus-host disease, macrophage functional heterogeneity, *in vitro* models of Hk (haemopoietic histocompatibility) mediated resistance and the control of allograft rejection and G-v-H disease. As such, the book is a "must" for those working in experimental marrow transplantation and viral leukaemogenesis (similar effector mechanisms seem to be invoked in resistance to marrow transplants and leukaemia cell grafts). However, it may be of only limited value to students or new entrants into the field of "natural resistance" mechanisms, since the nature of the discussions presume a rather wide knowledge of this field.

T. M. Dexter